# Genomic epidemiology of carbapenemase-producing *Enterobacter cloacae* complex in Argentina: a retrospective analysis (2016–2022)

**DOI:** 10.1128/spectrum.03471-25

**Published:** 2026-07-16

**Authors:** Juan Manuel de Mendieta, María Alejandra Menocal, María Belén Sanz, Denise De Belder, Melina Rapoport, Tomas Poklepovich, Fernando Pasteran, Alejandra Corso, Sonia Alejandra Gomez

**Affiliations:** 1 Servicio Antimicrobianos, INEI-ANLIS “Dr. Carlos G. Malbrán,” National and Regional Reference Laboratory in Antimicrobial Resistance (NRRLAR) 62986 , Ciudad Autónoma de Buenos Aires, Argentina; 2 Consejo Nacional de Investigaciones Científicas y Técnicas (CONICET) 161609, Ciudad Autónoma de Buenos Aires, Argentina; 3 ANLIS “Dr. Carlos G. Malbrán,” Unidad Operativa–Centro Nacional de Genómica y Bioinformática 62873, Ciudad Autónoma de Buenos Aires, Argentina; Universidade de Sao PauloMicrobiology, São Paulo, Brazil

**Keywords:** *Enterobacter* spp., antimicrobial resistance, carbapenemase, taxonomy, phylogenetic analysis, genomics, surveillance study

## Abstract

*Enterobacter* spp., after *Klebsiella pneumoniae*, is among the most common hospital-acquired enterobacteria associated with antimicrobial resistance (AMR) worldwide, yet it remains underexplored. We determined the taxonomic and epidemiological distribution of 131 representative carbapenemase-producing *Enterobacter* spp. recovered from infections across Argentina between 2016 and 2022. The sample included one isolate per patient from 17 jurisdictions and 68 hospitals. Outbreak-related and surveillance isolates were excluded. Sequencing was performed using Illumina, and bioinformatic analyses were used to determine species (average nucleotide identity [ANI] ≥94%) and subspecies (ANI ≥98%) using BactInspector/fastANI. Sequence types (STs) were assigned by multilocus sequence typing (MLST) via PubMLST, and resistance genes were identified using AMRfinderPlus/Resfinder. Samples were obtained from (*n*; %) urine (43; 33%), blood (41; 31%), and other sites (47; 36%). Seven species were identified, with *Enterobacter hormaechei* (EH) predominating ( 120; 92%) and comprising five subspecies. *E. hormaechei* subsp. *steigerwaltii* was the most abundant (60; 45%), followed by *E. hormaechei* subsp. *xiangfangensis* (29; 22%). *Enterobacter cloacae* subsp. *cloacae* was underrepresented (3; 2.3%). KPC-2 was the predominant carbapenemase, followed by NDM-1 and OXA-163. A total of 58 STs were identified, 38 represented by a single isolate. EH included 50 STs, with the most abundant being ST88 (12; 9%), ST78 (12; 9%), and ST66 (8; 6%). No clear associations were observed between species/subspecies, clones, and carbapenemase types. Overall, the population structure of carbapenemase-producing *Enterobacter* spp. in Argentina is complex and diverse, characterized by multiple species, subspecies, clonal diversity, and varied carbapenemase types and combinations. Notably, 50% of the isolates belonged to *E. hormaechei* subsp*. steigerwaltii*, suggesting a major role of this subspecies in the dissemination of AMR.

**IMPORTANCE:**

Carbapenemase-producing Enterobacter spp. rank as the most common Enterobacterales associated with carbapenemase production. Despite their clinical relevance, the taxonomy and population structure of this group remain poorly characterized. This study provides a comprehensive genomic overview of clinical carbapenemase-producing *Enterobacter* spp. circulating in Argentina, revealing a highly diverse population composed of species, subspecies, and clonal types, harboring mainly KPC-2, followed by NDM-1 and combinations. We identified international clones within *E. hormaechei*, such as ST88 and ST78, which may play important roles in the dissemination of antimicrobial resistance. Notably, 50% of the isolates belonged to *E. hormaechei* subsp. *steigerwaltii*, suggesting a major role of this subspecies in the dissemination of antimicrobial resistance (AMR). By improving our understanding of this complex bacterial group, this work supports the strengthening of antimicrobial resistance surveillance networks and the development of targeted strategies for infection control and antibiotic stewardship in healthcare settings.

## INTRODUCTION

The genus *Enterobacter* comprises facultative anaerobic, non-spore-forming, rod-shaped gram-negative bacilli belonging to the order *Enterobacterales* (1). This group is commonly found in various environments like fresh water, soils, and in the microbiota of plants and animals (2). In particular, this genus has been progressively recognized as a nosocomial pathogen, responsible for causing resistant infections, which is why it was included in the ESKAPE consortium (*Enterococcus faecium, Staphylococcus aureus, Klebsiella pneumoniae, Acinetobacter baumannii, Pseudomonas aeruginosa,* and *Enterobacter* spp.) (3). Furthermore, *Enterobacter* spp. has been included in the WHO global priority pathogens list of antibiotic-resistant bacteria (2024) as part of the *Enterobacterales* group, with carbapenem resistance as “Priority 1” critical group, representing the highest threat to public health due to limited treatment options, high disease burden, and increased resistance (4).

Currently, at least 28 different species and various subspecies of *Enterobacter* have been identified with valid and correct names (5, 6). The *Enterobacter cloacae* complex (ECC) is a subgroup within this genus that exhibits >60% DNA-DNA hybridization with *E. cloacae* species and includes *E. cloacae*, *Enterobacter hormaechei*, *Enterobacter asburiae*, *Enterobacter kobei*, *Enterobacter ludwigii*, *Enterobacter mori*, and *Enterobacter nimipressuralis*, with *E. cloacae* and *E. hormaechei* being the most reported species in hospital-acquired infections (2, 7). The List of Prokaryotic names with Standing in Nomenclature presently lists five validated subspecies of *E. hormaechei: E. hormaechei* subsp. *steigerwaltii*, *E. hormaechei* subsp. *xiangfangensis, E. hormaechei* subsp. *hoffmannii*, *E. hormaechei* subsp. *oharae*, and *E. hormaechei* subsp. *hormaechei* (https://lpsn.dsmz.de/species/enterobacter-hormaechei).

Recent surveillance studies indicate that *Enterobacter* spp. frequently rank as the second or third most common *Enterobacterales* associated with carbapenemase production (8). Moreover, carbapenem-resistant *Enterobacter* spp. ranks 12^th^ among bacterial pathogens of public health importance, published in the 2024 WHO priority pathogens list (4). In addition, multiple studies report the production of carbapenemases as the most predominant mechanism associated with carbapenem-resistant ECC (2, 9, 10).

The epidemiology of carbapenem-resistant *Enterobacterales* in Argentina paralleled regional and global trends. Recent studies showed an increased detection of carbapenem-resistant *Enterobacterales* in Asia Pacific, Europe, Latin America, and Middle East-Africa regions, mostly due to the widespread dissemination of *Klebsiella pneumoniae* carbapenemase (KPC) and New Delhi metallo-β-lactamase (NDM)-type carbapenemases, in addition to the OXA-type enzymes (10). This complex scenario is particularly concerning in Latin America, where resistance levels rose above those of other regions between 2020 and 2022. This increase was marked by the detection of previously less frequent species producing Class A carbapenemases, as well as the increasing co-expression of two or more carbapenemases (8, 11, 12).

In Argentina, the epidemiological landscape that emerged as a consequence of the COVID-19 pandemic was characterized by the equivalent circulation of NDM and KPC, primarily attributed to *K. pneumoniae*, followed by *Morganellaceae* species and ECC, among others (13). Particularly, the phenotypic profile of ECC has shown a steady increase in resistance to imipenem since the pandemic, ranging between 11% in 2020 and 14% in 2024, according to the data obtained from Argentina’s National Antimicrobial Resistance Surveillance system (World Health Organization Network, WHONET) (12–14).

Given the importance of ECC as a primary cause of disease and a driver of antimicrobial resistance (AMR) in hospital settings, the misidentification of *Enterobacter* species has become a significant concern for clinical microbiology laboratories and surveillance programs (2). Consequently, we performed a retrospective study to analyze the taxonomic distribution, subspecies composition, circulating clones, and carbapenemase content of isolates collected as part of the national surveillance of carbapenem-resistant and difficult-to-detect AMR mechanisms in gram-negative pathogens from Argentina, conducted by the National and Regional Reference Laboratory in Antimicrobial Resistance (NRRLAR).

## RESULTS

### Preliminary species ID and antimicrobial susceptibility testing results

Biochemical ID confirmed *E. cloacae* in the 61 tested isolates (see Table S1 at https://github.com/sgomez-atb/Supplementary-Material-for-Genomic-Epidemiology-of-CP-ECC-in-Argentina-2016-22-). These isolates were referred in 2016 (*n* = 28) and 2017 (*n* = 12), a period in which this method was the primary strategy for identifying *Enterobacterales* at the NRRLAR. Matrix-assisted laser desorption/ionization time-of-flight (MALDI-TOF) identification gradually replaced biochemical testing; therefore, most of the isolates identified by MALDI-TOF were referred to the NRRLAR during the 2019–2021 period. MALDI-TOF MS was run for 70 isolates, and the results were as follows (*n*; score range): *E. cloacae* (*n* = 65; 1.87–2.43); *E. xianfangensis* (*n* = 4; 2.17–2.37); and *Enterobacter bugandensis* (*n =* 1; 2.34).

Details on the susceptibility testing results can be found in Table S1 at https://github.com/sgomez-atb/Supplementary-Material-for-Genomic-Epidemiology-of-CP-ECC-in-Argentina-2016-22-. Briefly, all isolates showed a high level of non-susceptibility (NS, intermediate plus resistant categories) to beta-lactam antibiotics, including cephalosporins (>92% NS), the monobactam aztreonam (85.6% NS), and carbapenems (ertapenem, 92% NS; imipenem, 82% NS; and meropenem, 75.6% NS) (see Table S1 at https://github.com/sgomez-atb/Supplementary-Material-for-Genomic-Epidemiology-of-CP-ECC-in-Argentina-2016-22-). In addition, considerable resistance was detected for piperacillin-tazobactam (100% NS), ciprofloxacin (88% NS), trimethoprim-sulfamethoxazole (85.7% NS), and the aminoglycosides gentamicin (75% NS) and amikacin (60% NS). The drugs tested with potential treatment capacity were minocycline (30% NS), ceftazidime-avibactam (20% NS), fosfomycin (9.9% NS), and colistin (9.9% NS), which were shown to be the most active drugs.

### Genomic analysis of ECC: species determination by ANI

**Fig 1:**
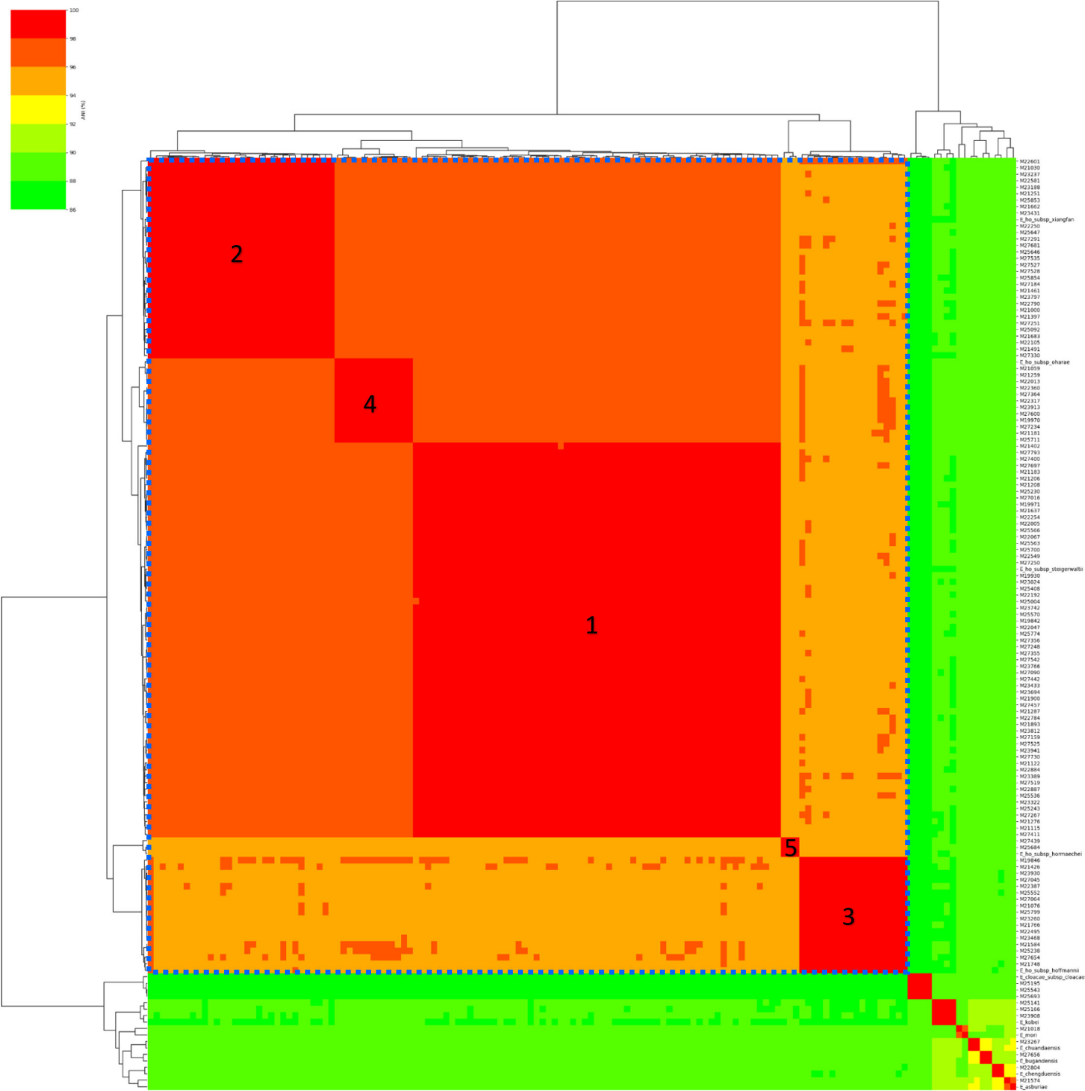
ANI cluster map of species and subspecies detected in this study. The dotted blue box line delimits *E. hormaechei* (ANI ≥94%) isolates. The five red squares within *E. hormaechei* represent the five subspecies detected (ANI ≥98%), numbered in decreasing cluster size order as follows: (1) *E. hormaechei* subsp. *steigerwaltii, n* = 60; (2) *E. hormaechei* subsp. *xiangfangensis, n* = 29; (3) *E*. *hormaechei* subsp. *hoffmannii*,* n* = 17; (4) *E. hormaechei* subsp. *oharae, n* = 12; (5) *E. hormaechei* subsp. *hormaechei*, *n = 2*. See text for more details. Besides *E. hormaechei,* seven clusters represented in small red boxes were identified, representing minor species of the ECC complex, including *Enterobacter cloacae*. Each line shows the ANI for each isolate with respect to the rest, including reference strains. Minor clusters comprised non-*hormaechei* isolates.

**Fig 2:**
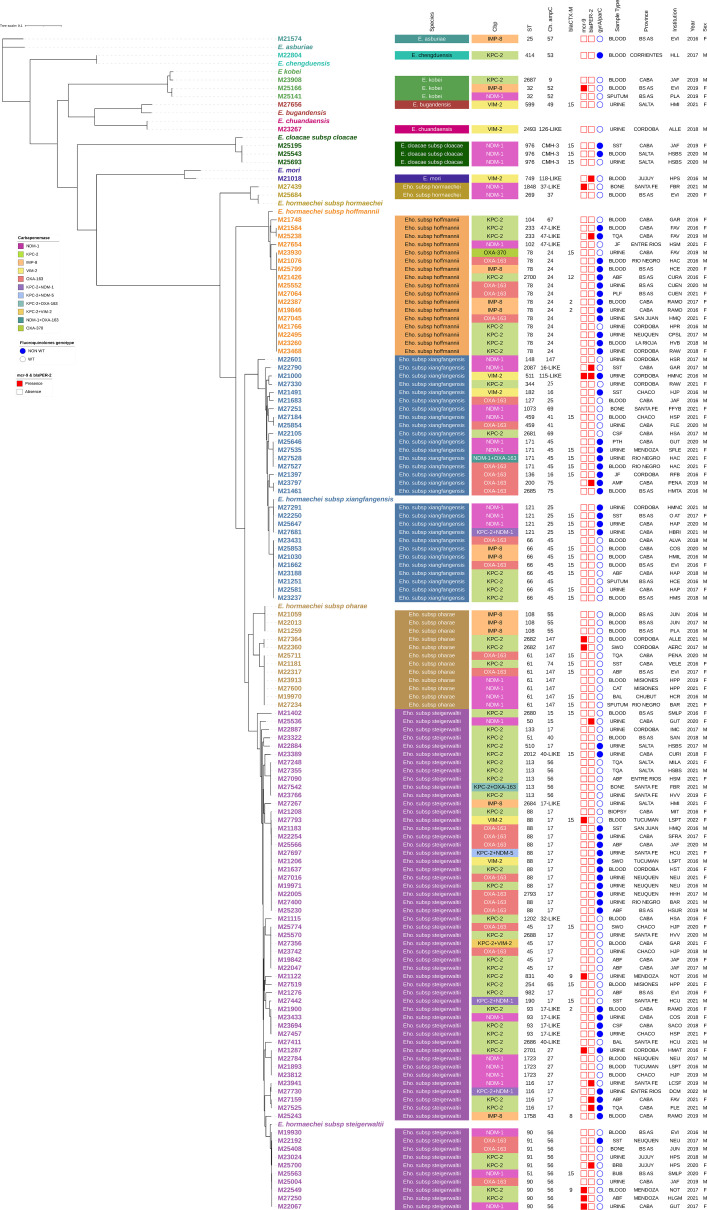
Phylogenetic tree of 131 *Enterobacter cloacae* complex isolates from infections in Argentina (2016–2022). Branch lengths are scaled to substitutions per site, inferred from a 447,070-base single-nucleotide polymorphism alignment derived from 1,882 core genes. The tree was annotated from left to right with species/subspecies, carbapenemase type and allele, sequence type, chromosomal *ampC*, *bla*_CTXM_ variant, presence/absence of *mcr-9* and *bla*_PER-2_, *gyrA*/*parC* genotype, sample type, province, institution, year of isolation, and patient’s sex. Cbp, carbapenemase; ST, sequence type; Ch., chromosomal; SST, skin and soft tissue; TQA, tracheal aspirate; JF, joint fluid; ABF, abdominal fluid; PLF, pleural fluid; CSF, cerebrospinal fluid; PTH, prosthesis; AMF, amniotic fluid; SWO, surgical wound; CAT, catheter; and BAL, bronchoalveolar lavage.

Average nucleotide identity (ANI) analysis confirmed *E. hormaechei* as the major group (120/131, 91.6%) (Fig. 1; see Table S2b at https://github.com/sgomez-atb/Supplementary-Material-for-Genomic-Epidemiology-of-CP-ECC-in-Argentina-2016-22-). This species was further clustered into five subspecies (*n*, %): *E. hormaechei* subsp. *steigerwaltii* (60, 45.8%), *E. hormaechei* subsp. *xiangfangensis* (29, 22.1%), *E. hormaechei* subsp. *hoffmannii* (17, 13%), *E. hormaechei* subsp. *oharae* (12, 9.2%), and *E. hormaechei* subsp. *hormaechei* (2, 1.5%) (Fig. 2). The remaining non-*E*. *hormaechei* species with lower representation were *E. kobei* (3, 2.3%), *E. cloacae* subsp. *cloacae* (3, 2.3%), *E. mori* (1, 0.8%), *Enterobacter chuandaensis* (1, 0.8%)*, E. bugandensis* (1, 0.8%)*, Enterobacter chengduensis* (1, 0.8%), *and E. asburiae* (1, 0.8%).

### Comparison of ID results obtained by MALDI-TOF and WGS

Comparison between the identification obtained by MALDI-TOF (*n* = 70) and whole-genome sequencing (WGS) (*n* = 131) revealed significant differences in taxonomic assignment within the *E. cloacae* complex (see Tables S1 and S2c at https://github.com/sgomez-atb/Supplementary-Material-for-Genomic-Epidemiology-of-CP-ECC-in-Argentina-2016-22-). Among the 65 isolates identified as *E. cloacae* by MALDI-TOF, only three were assigned as *E. cloacae* subsp. *cloacae* by WGS. The rest of the *E. cloacae* complex isolates identified by MALDI-TOF (*n* = 61) were reclassified by genomics as follows: *E. hormaechei* subspecies *steigerwaltii* (*n* = 35), *xiangfangensis* (*n* = 17), *oharae* (*n* = 4), *hoffmannii* (*n* = 4), and *E. hormaechei* subsp. *hormaechei* (*n* = 1), as described above. One isolate recognized as *E. cloacae* by MALDI-TOF (score, 2.12) was identified as *E. chuandaensis* by WGS. In addition, four isolates identified as *E. xiangfangensis* by MALDI-TOF (score range, 2.17–2.37) were reclassified by WGS into four *E. hormaechei* subsp.: *hormaechei, xiangfangensis, steigerwaltii*, and *oharae*. One isolate was successfully identified as *E. bugandensis* by both methods, MALDI-TOF (score, 2.34) and WGS. Of note, as mentioned in Materials and Methods, the rest of the isolates (*n* = 61) were identified by biochemical methods and WGS.

### Genomic analysis of resistance gene content

The 131 carbapenemase-producing *E. cloacae* complex (CP-ECC) isolates studied harbored 11 carbapenemase types or combinations (see Tables S3 and S2d at https://github.com/sgomez-atb/Supplementary-Material-for-Genomic-Epidemiology-of-CP-ECC-in-Argentina-2016-22-). The prevalent carbapenemase gene was *bla*_KPC-2_, followed by *bla*_NDM-1_. Their distribution was as follows: *bla*_KPC-2_ (49; 37.3%), *bla*_NDM-1_ (29; 22.1%), *bla*_OXA-163_ (26; 19.8%), *bla*_IMP-8_ (12; 9.2%), *bla*_VIM-2_ (7; 5.3%), and *bla*_OXA-370_ (1; 0.8%). Isolates co-producing multiple carbapenemases accounted for 5.3% (7/131) with the following distribution: *bla*_KPC-2_ + *bla*_NDM-1_ (3; 2.3%), KPC-2 + NDM-5 (1; 0.8%), KPC-2 + OXA-163 (1; 0.8%), KPC-2 + VIM-2 (1; 0.8%), and NDM-1 + OXA-163 (1; 0.8%) (Fig. 2; see Tables S1 and S2d at https://github.com/sgomez-atb/Supplementary-Material-for-Genomic-Epidemiology-of-CP-ECC-in-Argentina-2016-22-).

Among extended-spectrum beta-lactamase (ESBL)-producing ECC isolates, 37.4% (49/131) carried at least one ESBL gene, of which *bla*_CTX-M_ was the most frequent (38, 29%). In particular, *bla*_CTX-M-15_ was the predominant allele detected (*n* = 31; 23.7%) (Fig. 2). Other ESBLs were less common, including *bla*_PER-2_ (*n* = 10; 7.6%) and *bla*_SHV-7_ and *bla*_SHV-12_ (*n =* 3 each; 2.3%). Narrow-spectrum beta-lactamases were also found, primarily *bla*_TEM-1_ (*n* = 89; 67.9%) and *bla*_OXA-1_ (*n* = 68; 51.9%) (Fig. 2; see Tables S1 and S3 at https://github.com/sgomez-atb/Supplementary-Material-for-Genomic-Epidemiology-of-CP-ECC-in-Argentina-2016-22-).

As expected, nearly all isolates carried the chromosomal AmpC *bla*_ACT_, except for three *Enterobacter cloacae* subsp. *cloacae* isolates, which possessed the chromosomal AmpC *bla*_CMH_ (Fig. 2; see Tables S1 and S3 at https://github.com/sgomez-atb/Supplementary-Material-for-Genomic-Epidemiology-of-CP-ECC-in-Argentina-2016-22-). Three *E. cloacae* subsp. *cloacae* isolates harbored CMH-3. A total of 35 distinct AMP-C allelic variants were identified; *bla*_ACT-17_ was the most abundant (27; 20.4%), followed by *bla*_ACT-56_ (15, 11.1%) (Fig. 2; see Tables S1 and S3 at https://github.com/sgomez-atb/Supplementary-Material-for-Genomic-Epidemiology-of-CP-ECC-in-Argentina-2016-22-). Additionally, 16 isolates contained 11 novel *bla*_ACT_ variants. In these isolates, the bioinformatics programs and databases used, such as AMRfinder and ResFinder, could not assign a specific allele variant. Therefore, the closest variant was assigned. For these reasons, “new” variants were presumed.

Additional resistance determinants were also detected and detailed in Table S3 at https://github.com/sgomez-atb/Supplementary-Material-for-Genomic-Epidemiology-of-CP-ECC-in-Argentina-2016-22-. The accompanying resistance determinants included mechanisms across 11 antimicrobial families, besides beta-lactams, such as aminoglycosides, quinolones, phenicols, macrolides, and trimethoprim-sulfamethoxazole, among others. The mechanisms detected were the following: mentioned in decreasing order of abundance (mechanisms [*n*; %]): *oqxAB* (121; 92.4%), *dfrA* (103; 78.6%), *fosA* (94; 71.8%), *sul1* (93; 71%), *aadA1* (73; 55.7%), *aac(6′)-Ib-cr* (73; 55.7%), *mph*(A) (57; 43.5%), *catA1* (47; 35.9%), *parC*_S80I (45; 34.4%), *qnrB1* (29; 22.1%), and *tet*(A) (29; 22.1%). On average, each isolate contained 16 acquired and chromosomal AMR genes or point mutations.

The mobile-colistin resistance determinant *mcr-9* was identified in 11 isolates ([10]; 8.3%) (Fig. 2; see Tables S1 and S3 at https://github.com/sgomez-atb/Supplementary-Material-for-Genomic-Epidemiology-of-CP-ECC-in-Argentina-2016-22-). These included nine isolates harboring the *mcr-9.1* allele, and two harbored *mcr-9.2* (Fig. 2; see Table S1 at https://github.com/sgomez-atb/Supplementary-Material-for-Genomic-Epidemiology-of-CP-ECC-in-Argentina-2016-22-).

As detailed above, ECC isolates were phenotypically resistant to more than three antimicrobial families, confirming the multi-drug-resistant phenotype. It is worth mentioning that susceptibility testing was focused on the main antimicrobial families whose phenotype can be used for detection, epidemiological purposes, or treatment. Then, regardless of the presence of the gene that confers resistance to a particular drug, phenotypic testing was not performed for tetracyclines, macrolides-lincosamides-sulfonamides, and phenicols, as these drugs are not used in infections caused by these organisms.

### Clonal distribution of carbapenemase-producing ECC from Argentina

Multilocus sequence typing (MLST) results demonstrated a great clonal diversity among *Enterobacter* spp. clinical isolates (Fig. 2; see Table S3 at https://github.com/sgomez-atb/Supplementary-Material-for-Genomic-Epidemiology-of-CP-ECC-in-Argentina-2016-22-). A total of 58 STs were identified, and in particular, ST78 in *E. hormaechei* subsp. *hoffmannii* (*n* = 12/17) and ST88 in *E. hormaechei* subsp. *steigerwaltii* (*n* = 12/60) were the predominant STs, followed by ST66 in *E. hormaechei* subsp. *xiangfangensis* (*n* = 8/29) and ST61 in *E. hormaechei* subsp. *oharae* (*n* = 7/12). As expected, 50 of 58 STs were identified within the *E. hormaechei* isolates, considering the abundance of this species, and 25 STs among 60 isolates were found in *E. hormaechei* subsp. *steigerwaltii*. Additionally, 38 STs were identified in individual isolates. In all cases, all STs were associated with three or four different carbapenemases and were isolated during the full studied period (2016–2022) with a few exceptions: the five ST113 isolates were detected in 2021, and the four ST171 isolates were detected in 2020–2021 (Fig. 2).

The two major clonal types, ST78 *E. hormaechei* subsp. *hoffmannii* and ST88 in *E. hormaechei* subsp. *steigerwaltii*, showed broad dissemination between 2016 and 2022, considering they were present across the country in seven and eight jurisdictions, respectively (Fig. 2). In addition, ST78 and ST88 were associated with four distinct mechanisms as follows: ST78: IMP-8, OXA-163, OXA-370, and KPC-2, while ST88: KPC-2, OXA-163, VIM-2, and the double carbapenemase producer M27697 KPC-2 + NDM-5 (Fig. 2). In particular, the two ST88 VIM-2 isolates from Tucumán, M21206 and M27793, were obtained from the same hospital in 2016 and 2022, respectively. Both isolates differed in that the isolate obtained in 2022 (M27793) carried, in addition to *bla*_VIM_, *mcr-9, bla*_CTX-M-15_, and non-wild-type *gyrA*. These results suggest that ST88 has been present in the hospital for many years and that this clone acquired additional resistance determinants over time.

The other two relevant clonal types were ST66 in *E. hormaechei* subsp. *xiangfangensis* (*n* = 8) and ST61 in *E. hormaechei* subsp. *oharae* (*n* = 7), both detected between 2016 and 2020 (Fig. 2). Both clones were highly associated with *bla*_CTX-M-15_ (*n* = 5 in each ST). ST66 was recovered exclusively in Buenos Aires City and Province, while ST61 was detected in five jurisdictions geographically distant from one another: Río Negro and Chubut (Patagonia), Misiones (North), and Buenos Aires City and Province (Center). ST66 was associated with *bla*_OXA-163_, *bla*_IMP-8_, and *bla*_KPC-2_, while ST61 was associated mainly with *bla*_NDM-1_ (*n* = 4), *bla*_OXA-163_, and *bla*_KPC-2_.

Minor clones were ST113 (*n* = 5), ST45 (*n* = 5), ST90 (*n* = 5), ST116 (*n* = 4), ST91 (*n* = 4), and ST93 (*n* = 4) in *E. hormaechei* subsp. *steigerwaltii*. While ST121 and ST171 (*n* = 4, each) were detected in *E. hormaechei* subsp. *xiangfangensis*. As mentioned above, these clones were recovered throughout the study period and showed broad geographical distribution and diverse resistance gene content, as detailed in Fig. 2 and see Table S3 at https://github.com/sgomez-atb/Supplementary-Material-for-Genomic-Epidemiology-of-CP-ECC-in-Argentina-2016-22-. Three of five ST90 harbored *mcr-9,* where M22549 and M27250 harbored KPC-2 plus the *mcr-9.2* variant. Both were isolated in different institutions in Mendoza Province in 2017 and 2021, respectively, confirming that there was no epidemiological link between them.

The three *E. cloacae* subsp. *cloacae* of our sample belonged to ST976 and were NDM-1 and CTX-M-15 producers, obtained in 2019 and 2020 in Buenos Aires City and the northern province of Salta, respectively (Fig. 2). Finally, an *E. mori* ST749 (M21018) was recovered in 2016 from an infection in a hospital in the Northern Province of Jujuy as a VIM-2 plus PER-2 producer. ST749 was reported by our group for the first time in 2012 as novel, which had been obtained from a VIM-2 plus PER-2-producing *E. cloacae* obtained from a bacteremia in the same province, suggesting the endurance of this ST in the hospital environment for all these years (15).

### Analysis of plasmid content in ECC

The plasmid content across isolates was diverse, with the number of replicons per isolate ranging from 1 to 10 (median = 4) (see Table S4 at https://github.com/sgomez-atb/Supplementary-Material-for-Genomic-Epidemiology-of-CP-ECC-in-Argentina-2016-22-). The most frequent number of replicons per isolate was four (*n* = 51), five (*n* = 22), and six (*n* = 17). The detected plasmids belonged primarily to 10 families, including Col, IncA, IncC, IncF, IncH, IncM, IncN, IncP, IncQ, and IncX, among others.

The plasmid replicon types and their association with antimicrobial resistance genes, particularly carbapenemases, were analyzed (see Table S4 at https://github.com/sgomez-atb/Supplementary-Material-for-Genomic-Epidemiology-of-CP-ECC-in-Argentina-2016-22-). The prevalent replicon was Col(pHAD28), detected in 94 of 131 isolates (71.8%); in 25 of these isolates, it was associated with the *qnrB19* gene. After Col(pHAD28), IncM1 and IncHI2 were the most frequently identified replicons (see Table S4 at https://github.com/sgomez-atb/Supplementary-Material-for-Genomic-Epidemiology-of-CP-ECC-in-Argentina-2016-22-).

Notably, IncM1 was detected in 34 KPC-2-producing isolates (62%), distributed across 8 jurisdictions, 23 sequence types, 3 species (*E. hormaechei*, *E. chengduensis*, and *E. kobei*), and all *E. hormaechei* subspecies except *E. hormaechei* subsp. *hormaechei* (see Table S4 at https://github.com/sgomez-atb/Supplementary-Material-for-Genomic-Epidemiology-of-CP-ECC-in-Argentina-2016-22-). In 16 of the 34 isolates, IncM1 was located on the same contig as the *bla*_KPC-2_ gene, confirming the association. These findings suggest that the IncM1-*bla*_KPC-2_ platform has a high capacity to reach broad geographic, interspecies, and inter-clonal distribution.

Plasmid analysis of the 34 NDM-producing isolates (33 NDM-1 and 1 NDM-5) showed that the IncC replicon was the prevalent (*n* = 27; 79.4%) (see Table S4 at https://github.com/sgomez-atb/Supplementary-Material-for-Genomic-Epidemiology-of-CP-ECC-in-Argentina-2016-22-). This replicon was detected across 11 jurisdictions and 17 sequence types in *E. kobei* and in all subspecies of *E. hormaechei*. In 23 of the 27 isolates, IncC was located on the same contig as *bla*_NDM-1_. These findings indicate a predominant role of IncC plasmids in the geographic spread and interclonal dissemination of the *bla*_NDM-1_ gene.

Among the *bla*_OXA-48-like_-producing isolates (28 OXA-163 and 1 OXA-370), the prevalent replicon was Col(pHAD28) (*n* = 21; 72.4%), followed at similar frequencies by IncHI2, IncHI2A, IncM1, and IncFII(Yp) (see Table S4 at https://github.com/sgomez-atb/Supplementary-Material-for-Genomic-Epidemiology-of-CP-ECC-in-Argentina-2016-22-). A clear genetic association between a replicon and *bla*_OXA-48-like_ was demonstrated in only three isolates, in which replicon IncC was located on the same contig as the resistance gene, limiting our ability to determine the principal plasmid vehicles involved in its dissemination. Although these three isolates originated from the same jurisdiction (CABA), they belonged to three different sequence types and three distinct subspecies (*E. hormaechei* subsp. *xiangfangensis*, subsp. *steigerwaltii*, and subsp. *oharae*), suggesting that dissemination may not be restricted to a single clonal background.

In the VIM-2-producing (*n* = 8) and IMP-8-producing (*n* = 12) isolates, the contigs carrying the respective carbapenemase genes could not be linked to a specific plasmid replicon type. Among the VIM-2 producers, IncN3 was the predominant replicon (*n* = 7; 87.5%), whereas Col(pHAD28) predominated among the IMP-8 producers (*n* = 9; 75%), followed by IncFII (*n* = 7; 58%) and IncM1 (*n* = 7; 58%) (see Table S4 at https://github.com/sgomez-atb/Supplementary-Material-for-Genomic-Epidemiology-of-CP-ECC-in-Argentina-2016-22-).

Overall, in cases where carbapenemase genes were not located on the same contigs as identified plasmid replicons, a direct genetic linkage could not be confirmed, and complete plasmid reconstruction would be necessary to accurately determine the replicon platforms involved in their dissemination.

### Close genetic environment of carbapenemase genes

The analysis of the close environment of *bla*_KPC-2_ showed that it was associated with the Tn*4401a* transposon in only two isolates (see Table S3 and Fig. S2 at https://github.com/sgomez-atb/Supplementary-Material-for-Genomic-Epidemiology-of-CP-ECC-in-Argentina-2016-22-). In the rest of the isolates, *bla*_KPC-2_ was found in the ΔTn*3*-derived transposon, a configuration typically referred to as non-Tn*4401* element (NTE_KPC_) (*n* = 53; 95.9%) (Table S3) (16). Among the NTE_KPC_, in the majority of the isolates, the configuration was as follows: IS*Kpn27-bla*_KPC-2_-ΔIS*Kpn6*, followed by the typical structure, including Δ*bla*_TEM_: IS*Kpn27*-Δ*bla*_TEM_-*bla*_KPC-2_-ΔIS*Kpn6*, detailed in Table S3 and Fig. S2 at https://github.com/sgomez-atb/Supplementary-Material-for-Genomic-Epidemiology-of-CP-ECC-in-Argentina-2016-22- (16).

The close genetic environment of *bla*_NDM-1_ rendered two main groups: one associated with *rmtC* and another without *rmtC* (see Table S3 and Fig. S2 at https://github.com/sgomez-atb/Supplementary-Material-for-Genomic-Epidemiology-of-CP-ECC-in-Argentina-2016-22-). In all cases, the gene order downstream of *bla*_NDM-1_ showed the typical conserved arrangement comprising *bla*_NDM-1_-*ble-trpF-tat-dct*. The *rmtC* isolates (19/34; 56%) harbored *bla*_NDM-1_ in association with an environment previously described in Argentina with the following gene order: *rmtC-*ΔIS*Ecp1*-IS*Kpn14*-ΔIS*Aba125-bla*_NDM-1_-*ble*_MBL_*-trpF-tat-dct-groES-groEL*-ΔIS*CR27* (17). In the second group, *bla*_NDM-1_ was embedded in an identical or highly similar environment but without *rmtC* (14/34; 41%) (see Table S3 and Fig. S2 at https://github.com/sgomez-atb/Supplementary-Material-for-Genomic-Epidemiology-of-CP-ECC-in-Argentina-2016-22-). Finally, the close genetic environment of M27697, the only isolate harboring *bla*_NDM-5_, showed the gene order: ΔIS*Aba125*/IS30-*bla*_NDM-5_-*ble-trpF-tat-dct-*IS*91*. This environment is highly similar to that recently described in a *K. pneumoniae* ST307 co-producing *bla*_KPC-2_ and *bla*_NDM-5_ (GenBank accession: CP166596.1) in association with *rmtB1* in the upstream proximity (18, 19). Of note, M27697 was a double carbapenemase producer (KPC-2 and NDM-5) and also harbored *rmtB1*. However, we were unable to confirm a direct linkage between *rmtB* and *bla*_NDM-5_ due to sequencing limitations, as mentioned above (see Table S3 at https://github.com/sgomez-atb/Supplementary-Material-for-Genomic-Epidemiology-of-CP-ECC-in-Argentina-2016-22-).

In the genetic environment surrounding *bla*_OXA-163_ and *bla*_OXA-370_, all isolates harbored a truncated IS*4* fragment downstream of the gene (see Table S3 and Fig. S2 at https://github.com/sgomez-atb/Supplementary-Material-for-Genomic-Epidemiology-of-CP-ECC-in-Argentina-2016-22-). A remnant of a Tn*21*-like element was found upstream of the *bla*_OXA_ gene (27/29; 93%) (see Table S3 at https://github.com/sgomez-atb/Supplementary-Material-for-Genomic-Epidemiology-of-CP-ECC-in-Argentina-2016-22-), as previously reported (20). Additionally, the genetic configuration Δ*rhs2*-ΔTn*21*-IS*4321*-ΔTn*21*-like-*bla*_OXA-163-_ΔIS*4*-like was identified in four isolates (M21662, M22317, M23431, and M25711) (see Table S3 and Fig. S2 at https://github.com/sgomez-atb/Supplementary-Material-for-Genomic-Epidemiology-of-CP-ECC-in-Argentina-2016-22-).

The metallo-β-lactamase (MBL) genes *bla*_VIM-2_ and *bla*_IMP-8_ were found as first cassettes of class 1 integrons. In particular, six *bla*_VIM-2_ isolates were embedded as gene cassettes that match those of the In*900* with the following gene order: *bla*_VIM-2_-*aadA7* (see Table S2 at https://github.com/sgomez-atb/Supplementary-Material-for-Genomic-Epidemiology-of-CP-ECC-in-Argentina-2016-22-). In these cases, the 5′ CS and 3′ CS were either not detected or partially detected due to the sequencing approach, except in M27656, in which In900 was complete (see Table S3 at https://github.com/sgomez-atb/Supplementary-Material-for-Genomic-Epidemiology-of-CP-ECC-in-Argentina-2016-22-). In one isolate, M21491, *bla*_VIM-2_ was found in In*883* as follows: 5′ CS-*bla*_VIM-2_-*aacA4-aacA38-bla*_OXA-2_-*gcuDD3*-3′ CS. In the case of *bla*_IMP-8_, the gene order was detected in six isolates as follows: 5′ CS-*bla*_IMP-8_-*aadA1a-* retron-type RNA-directed DNA polymerase-like (see Table S3 at https://github.com/sgomez-atb/Supplementary-Material-for-Genomic-Epidemiology-of-CP-ECC-in-Argentina-2016-22-). This structure shared 100% sequence identity and 100% coverage with the sequence of an *Escherichia coli* from Argentina (GenBank accession: MG550958, unpublished). In the remaining six isolates, it was not possible to determine the complete structure of class 1 integrons in the assembled contigs obtained due to the sequencing approach.

### Susceptibility patterns against antimicrobials with potential treatment capacity

To understand the phenotypic behavior of ECC, focusing on those drugs with potential treatment capacity, we performed a detailed analysis of the percentages of non-susceptibility (intermediate plus resistant) to those drugs not affected by the enzymatic activity of the carbapenemases (Table 1). Briefly, colistin and fosfomycin exhibited the highest overall activity (90%) for all isolates, followed by minocycline (70%). Ceftazidime-avibactam (CZA) achieved an overall susceptibility rate of 58.8%, primarily due to the presence of metallo-β-lactamase producers, which accounted for 36.6% of the isolates. Within the KPC-producing group, CZA was the most active agent (98% susceptibility), with the remaining 2% resistance attributed to an isolate that coproduced an ESBL of the PER type. The next most active agents within KPC producers were fosfomycin (94%), colistin (92%), and minocycline (71.4%).

**TABLE 1:** Susceptibility profiles of antibiotics with potential treatment capacity stratified by carbapenemase class^*d*^

	No. of isolates	% Susceptibility								
		COL	FOS	MNO	CZA^*b*^	ATM	GEN	AKN	CIP	SXT
Total	131	90.1	90.1	70.2	96.1	14.4	25.2	39.7	11.9	14.3
Carbapenemase class										
A	49	91.8	93.9	71.4	98.0	NA	34.7	61.2	15.6	26.3
B	48	85.4	87.5	75.0	NA	27.7	16.7	25.0	10.6	15.2
D	27	96.3	85.2	66.7	92.6	0.0	25.9	29.6	5.3	5.3
A + B + D	7	85.7	100	42.9	100	NA	14.3	28.6	14.3	0
Species^*c*^										
*E. hormaechei^e^*	**120**	**95**	**92.5**	**68.3**	**96**	**11.1**	**26.7**	**40**	**12.1**	**14.6**
*E. hormaechei* subsp*. steigerwaltii*	60	90	95	73.3	97.7	2.6	28.3	46.7	18.9	17.9
*E. hormaechei* subsp*. xiangfangensis*	29	100	93.1	51.7	100	10	27.6	27.6	3.8	9.5
*E. hormaechei* subsp*. hoffmannii*	17	100	76.5	64.7	84.6	9.1	35.3	52.9	13.3	64.7
*E. hormaechei* subsp*. oharae*	12	100	100	92.0	100.0	44^*a*^	0.0	25.0	0.0	11.1

^*a*^ *P *< 0.001.

^*b*^ CZA was only evaluated in isolates carrying class A carbapenemases, class D carbapenemases, or a combination of classes A and D.

^*c*^ *E. hormaechei* subsp. *hormaechei* (*n *= 2) were not included in the analysis of susceptibility profiles because it was represented by only two isolates.

^*d*^ Class A, serine carbapenemases; B, metalloenzymes; D, oxacillinase enzymes. CIP, ciprofloxacin; SXT, trimethoprim/sulfamethoxazole; ATM, aztreonam; CZA, ceftazidime/avibactam; GEN, gentamicin; AKN, amikacin; MNO, minocycline; COL, colistin; FOS, fosfomycin; and NA, not applicable.

^*e*^ Bold numbers show suspectibility testing results of *E. hormaechei*, the major species, and include the two subspecies exclued in footnote “c”.

Among MBL producers, fosfomycin (87.5%) and colistin (85.4%) demonstrated the greatest activity, followed by minocycline (75%) (Table 1). Aztreonam, an agent unaffected by this carbapenemase class, showed a susceptibility rate of 27.7%. This high level of resistance likely reflects the co-production of AmpC and/or ESBL enzymes. For class D carbapenemase producers, colistin (96.3%) showed the highest susceptibility rate, followed by CZA (92.6%), fosfomycin (85.2%), and minocycline (66.7%). In contrast, agents such as trimethoprim-sulfamethoxazole and ciprofloxacin were the least active drugs, with susceptibility rates ranging from 5% to 26%. Regarding aminoglycosides, the MBL-producing group showed the lowest susceptibility values, both for amikacin (25%) and gentamicin (16.7%). A significant association was observed between the production of NDM and the presence of methyltransferases in comparison with KPC-producing Rmt isolates (*bla*_NDM_ = 33, *rmt* = 27; *bla*_KPC_ = 55, *rmt* = 4 [*P* < 0.0001]).

Multiple carbapenemase producers (*n* = 7) showed 100% susceptibility to fosfomycin, followed by colistin (85.7%) (Table 1). However, minocycline displayed 43% activity in this group, in contrast to the susceptibility rates observed in the other carbapenemase classes, ranging from 67% to 75%.

As mentioned above, colistin resistance was identified in 13 isolates, of which only two (M22067 and M25166) carried *mcr-9.1*. Notably, seven of the *mcr-9.1*-positive isolates remained phenotypically susceptible to colistin, while M22067 and M25166 were resistant. It is important to note that the presence of *mcr-9* does not invariably result in phenotypic resistance. Its expression appears to be regulated by colistin induction in conjunction with the adjacent *qseBC* two-component system (21). Here, we were unable to confirm the presence of these regulatory genes in the colistin-susceptible isolates as the *mcr* contigs were fragmented due to the sequencing method employed. In addition, colistin-resistant isolates were checked for the presence of additional genetic determinants of colistin resistance, such as *pmr* mutations (e.g., *mgrB*, *phoP*, *phoQ*, *pmrA*, and *pmrB*) (2). Only *E. hormaechei*. subsp. *steigerwaltii* isolate (M27356) harbored an *mgrB* gene (negative regulator of *phoP/phoQ*) with a nucleotide deletion that produced a nonsense mutation and a nonfunctional protein.

We also aimed to identify differences in the susceptibility profiles among the major species, *E. hormaechei,* in response to ceftazidime-avibactam and aztreonam (ATM) (Table 1). The susceptibility results were consistent with the general drug profiles, and the observed phenotypes aligned with the detected carbapenemase genes. Specifically, *E. hormaechei* subsp. *steigerwaltii* demonstrated statistically higher susceptibility to CZA (73%; 44/60, *P* < 0.03) compared with *E. hormaechei* subsp. *xiangfangensis* and *E. hormaechei* subsp. *oharae*, explained by its higher KPC content (KPC, 30/60; Class B 13/60 [NDM = 9, VIM = 2, and IMP = 2]) (see Table S2b at https://github.com/sgomez-atb/Supplementary-Material-for-Genomic-Epidemiology-of-CP-ECC-in-Argentina-2016-22-). In contrast, *E. hormaechei* subsp. *xiangfangensis* showed 48% susceptibility to CZA, consistent with a higher Class B enzyme content (13/29, NDM = 9, VIM = 2, and IMP = 2) compared to Class A (KPC = 6). Similar patterns were observed for ATM, with lower susceptibility among subspecies harboring more KPC genes and higher susceptibility among those with higher Class B carbapenemase content. In particular, 7 of the 12 *E. hormaechei* subsp. *oharae* isolates carried a Class B enzyme (NDM-1 = 4 and IMP-8 = 3).

### Phylogenetic distribution of carbapenemase-producing ECC from Argentina

An overview of the assembled *Enterobacter* genome properties is provided in Table S5 at https://github.com/sgomez-atb/Supplementary-Material-for-Genomic-Epidemiology-of-CP-ECC-in-Argentina-2016-22-. The pan-genome obtained included a total of 33,265 genes, including both core and accessory genes. From this pan-genome, 1,882 core genes present in at least 99% of the studied isolates were identified. We constructed an alignment of these core genes spanning 1,902,702 nucleotides, and the resulting single-nucleotide polymorphism (SNP) alignment covered 447,070 bases.

The pan-genome phylogenetic tree distributed the eight identified species and their corresponding subspecies into distinct clusters (Fig. 2). Briefly, isolates formed separate branches, with the largest cluster corresponding to *E. hormaechei*, which was subdivided into five branches representing its subspecies. The remaining clades were divided into seven smaller branches, each representing different species. In agreement with the phylogenetic tree, species boundaries were defined using an ANI cutoff of 94%, and subspecies were delineated at 98% (Fig. 1).

## DISCUSSION

In this study, we conducted an in-depth genomic analysis of 131 carbapenemase-producing *Enterobacter* spp. clinical isolates collected between 2016 and 2022 across Argentina to characterize their taxonomic distribution, population structure, and genetic determinants of antimicrobial resistance. We identified seven species and six subspecies, with *E. hormaechei* subsp. *steigerwaltii* being the prevalent.

Originally, most of the carbapenemase-producing isolates described here were identified as *E. cloacae* or members of the *E. cloacae* complex using traditional biochemical and/or proteomic methods (see Tables S1 and S2c at https://github.com/sgomez-atb/Supplementary-Material-for-Genomic-Epidemiology-of-CP-ECC-in-Argentina-2016-22-). The application of WGS allowed for more detailed taxonomic discrimination, revealing that most of the isolates initially identified by MALDI-TOF as *E. cloacae* actually corresponded to *E. hormaechei,* primarily the subspecies *steigerwaltii* and *xiangfangensis*. Furthermore, WGS enabled the identification of less frequent species such as *E. chuandaensis*, among others. Continued genomic surveillance is warranted to monitor whether the less-represented species/subspecies increase over time or act as additional vehicles for carbapenemase spread. Although MALDI-TOF MS provides rapid and generally reliable identification at the genus and often species level—typically sufficient for routine clinical management—whole-genome sequencing offers the higher taxonomic resolution required for accurate AMR surveillance and epidemiological analyses (22).

The taxonomy of the *E. cloacae* complex, particularly *E. hormaechei*, is subject to ongoing genome-based revisions that may influence reported species prevalence depending on the ANI thresholds and reference genomes applied (5, 23, 24). In this study, species and subspecies were delineated using overall genome-related indices (OGRIs), specifically ANI, applying cutoffs of 94% for species and 98% for subspecies. This framework follows the criteria proposed by Chun et al. (25), whereby OGRI values between subspecies and other species remain below the species-level threshold, values among subspecies exceed this threshold, and each subspecies forms a genomically coherent and distinct clade in phylogenomic analyses (Fig. 1 and 2) (25). Our classifications were fully supported by phylogenetic reconstruction, demonstrating consistent clustering at both taxonomic levels. While future taxonomic refinements may adjust subspecies boundaries, ANI- and phylogeny-supported reconstructions, underscores its central role in the Argentinean epidemiology of clinical carbapenemase-producing *Enterobacterales* and highlights the importance of accurate genomic identification for ongoing surveillance and antimicrobial resistance monitoring.

In this study, *E. hormaechei* subsp. *steigerwaltii* was the dominant species, identified in nearly half of all isolates (60/131; ~46%). This finding highlights the significant role of this subspecies in the dissemination of carbapenem resistance in the country. *E. hormaechei* subsp. *steigerwaltii* was first described by Hofmann et al. (7, 26) based on the *hsp60* and corresponds to the genetic cluster VIII of the *E. cloacae* complex. It has been reported across multiple continents and is associated with resistance determinants such as IMP-8, IMP-14, VIM-1, NDM-1, KPC-2, and OXA-48 (27). In terms of clonal types in *E. hormaechei* subsp. *steigerwaltii*, this subspecies was represented by 25 different STs among 60 isolates, demonstrating significant clonal diversity. Notably, ST88 was the most abundant ST (12/60 isolates in eight jurisdictions) and not ST90 and ST93, as described by others (27–29). In this report, ST88 was mainly associated with *bla*_OXA-163_ (6/12 isolates), followed by *bla*_KPC-2_ (4/12 isolates) and *bla*_VIM-2_ (2/12 isolates). We previously reported the detection of ST88 in Buenos Aires City in 2011, marking the emergence of a chromosomally encoded *bla*_VIM-11_-In*346-*producing ECC strain (15). Here, the two VIM-producing ST88 isolates were obtained in one hospital of the northern province of Tucumán in 2016 and 2022. They only differed in their accessory resistance gene content (see below). Both isolates harbored *bla*_VIM-2_-In*900* instead of *bla*_VIM-11_-In*346*, demonstrating a progressive expansion and diversification of ST88 over time (15). To the best of our knowledge, ST88 has previously been reported to cause sporadic infections in China, in one case in Uruguay obtained from an *rmt*-harboring ECC from a rectal swab, and by our group, as mentioned above (15, 30, 31). Overall, ST88 appears to be a rare and infrequent clone in the literature, not fitting the “high-risk clone” definition, as detailed previously by Peirano et al. (32).

*E. hormaechei* subsp. *xiangfangensis* belongs to Hoffmann’s cluster VI (33). In our study, this subspecies was the second most abundant group (29/131, 13%), unlike other reports where it was found as the first subspecies (27, 29, 34). Again, this group also displayed broad clonal diversity (15 STs among 29 isolates) and harbored multiple carbapenemases, including KPC-2, NDM-1, IMP-8, OXA-163, and two double producers, KPC-2 + NDM-1 and NDM-1 + OXA-163. In Brazil, this subspecies was associated particularly with NDM-1 in ST136, emerging as a pathogen of companion animals (35). Nevertheless, ST66 was the most abundant clone here, distributed exclusively in Buenos Aires City and Province, followed by ST171, both mentioned in the literature with potential to become high-risk clones (36). Of note, an NDM-1-producing *E. hormaechei* subsp. *xiangfangensis* ST171 isolate obtained from a dog with a gall bladder infection in 2022 was recently reported by our group, demonstrating the circulation of this clone in the community and pets (37).

*E. hormaechei* subsp. *hoffmannii* was the third most abundant subspecies (17/131, 13%) and was associated with KPC-2, IMP-8, NDM-1, and two OXA-48-like variants, OXA-163 and OXA-370. In addition, most isolates in this group harbored *bla*_ACT-24_ and the non-wild-type *gyrA/parC*. This subspecies has been defined as clade D containing the Hoffmann cluster III type strain (2). In particular, the isolates studied here differed from the other subspecies in that 70% (12/17) belonged to the high-risk clone ST78 (36).

In this study, KPC-2 was the predominant carbapenemase type, followed by NDM-1, OXA-163, IMP-8, and VIM-2. These findings are similar to reports from the American continent, including the United States and Canada, but contrast with reports from Europe or Asia, where NDM and VIM-type enzymes are prevalent in ECC (27, 29, 38, 39). Altogether, it seems that the carbapenemase content in ECC follows regional variations. In this sense, the predominance of KPC in ECC in Argentina reflects the local epidemiology, where KPC has been endemic since shortly after the introduction of Tn*4401a-bla*_KPC-2_-producing *K. pneumoniae* ST258 (40). Over time, *bla*_KPC-2_ disseminated to other species, mostly associated with NTE_KPC_ configuration (16). Notably, *bla*_KPC-2_ in ECC was found embedded in the NTE_KPC_ configuration mostly without Δ*bla*_TEM_, which was previously the most frequent structure in Argentina (also called Variant 1a) (16, 40).

Regarding the dissemination of *bla*_KPC-2_, we identified a strong association with IncM1, detected across multiple jurisdictions, species, and sequence types. IncM1 belongs to the IncL/M plasmid family, historically and still commonly referred to as IncL/M, a group characterized as a broad host range and recognized as vehicles for carbapenemase dissemination (41). Although IncM1 is not among the most frequently described plasmid backbones, it has been associated with KPC, NDM, OXA-48, and ESBL genes, highlighting its role as an interspecies vehicle for life-threatening resistance genes (41). Locally, IncL/M-*bla*_KPC-2_ plasmids were reported by our group in 2010, coinciding with the emergence and dissemination of *K. pneumoniae* ST258 in Argentina (39). More recently, we described IncM1 plasmids harboring *bla*_KPC-2_ embedded in NTE_KPC_ in *K. pneumoniae* ST307, further supporting their persistence in the regional epidemiology (18). In South America, IncM1 has also been reported in Brazil in *K. pneumoniae* ST147 (42). Nevertheless, additional genomic studies, ideally including complete plasmid reconstruction, are needed to better define the stability, transmissibility, and epidemiological impact of IncM1 plasmids in *Enterobacter* spp.

In the case of NDM, this mechanism was first reported in our country in 2014, and since then, it has spread, gaining a leading position locally, mostly in *Enterobacterales* (12, 13, 43). The close genetic environment of *bla_NDM-1_* showed the typically conserved genes in the close proximity of *bla*_NDM_ with some variations, as reported previously (17, 18). Overall, the close environment of *bla*_NDMs_ was distinguished mostly by the presence or absence of a methylase enzyme of the *rmt* type, which confers high-level resistance to aminoglycosides (15).

Analysis of the plasmids associated with *bla*_NDM_ revealed that IncC was the prevalent replicon (see Table S3 at https://github.com/sgomez-atb/Supplementary-Material-for-Genomic-Epidemiology-of-CP-ECC-in-Argentina-2016-22-). This plasmid was identified nationwide across multiple clonal types and various species and subspecies of *Enterobacter*. IncC plasmids are conjugative, broad-range plasmids that have been circulating in Argentina since the emergence of NDM (17, 18). Our results confirm that these plasmids remain active drivers of NDM dissemination within *Enterobacter* spp. in clinical settings. IncC plasmids have a worldwide distribution and are found on all continents except Antarctica (44). These plasmids are characterized as type 1 and type 2, defined by specific hallmarks in the backbone and resistance island (ARI). In particular, IncC type 1 plasmids are found to mostly disseminate *bla*_NDM-1_, although in this study, this could not be confirmed due to the sequencing approach used. Of note, IncC and IncA plasmids were historically classified as “IncA/C” replicons; however, they were later confirmed to be compatible, abolishing each other’s entry into the cell (17).

The analysis of class D enzymes in our ECC collection revealed that almost 20% of isolates harbored *bla*_OXA-163_, a local and well-spread variant of *bla*_OXA-48_, in addition to the isolates expressing OXA-163 in combination with KPC or NDM (20). This particular variant was first described in 2011, and since then it has spread over the years mostly silently, to our best knowledge, only in Argentina and Egypt (20). This was confirmed in a recent prevalence study led by the NRRLAR, where 7.5% (61 out of 822) of carbapenem-resistant *Enterobacterales* isolates were *bla*_OXA-163_ producers (13). This dissemination can partly be explained by the fact that OXA-163 and emerging variants are difficult to detect by phenotypic, biochemical, and colorimetric methods. They express low hydrolytic capacity toward carbapenems and high hydrolytic power against extended-spectrum cephalosporins. In addition, OXA-163 is not inhibited by boronic acid, clavulanic acid, or sulbactam; instead, it is only inhibited by avibactam (20). As a result, our local network refers these isolates to the NRRLAR for molecular confirmation when the institution lacks additional confirmation methods (20). Different molecular studies suggest that *bla*_OXA-163_ may have originally been captured by mobile genetic elements from the chromosome of *Shewanella*, an aquatic bacterium, and was later integrated into plasmids. In this study, the close genetic environment of *bla*_OXA_ showed that 27 of 29 isolates harbored a remnant of a Tn*21*-like element upstream of the gene. This finding is in agreement with previous studies by our group (20). The presence of these conserved genetic markers previously reported suggests that *bla*_OXA-163/370_ may share a common, more ancient acquisition and mobilization event (20). Finally, the dissemination vehicles for *bla*_OXA-48-like_ genes were only identifiable in three isolates, all linked to IncC replicons, limiting our ability to characterize the specific plasmids driving the spread of these genes in *Enterobacter* spp.

During the early years of carbapenemase dissemination in Argentina, ECC was exclusively associated with VIM-2 (15). Briefly, we reported six VIM-producing *E. cloacae*, which represented all the VIM-producing *Enterobacterales* received from six institutions during 2008–2013 at the NRRLAR, as part of our surveillance (15). In this study, all isolates harbored *bla*_VIM-2_ in class 1 integron structures that, to our best, have only been reported by the NRRLAR in clinical carbapenemase-producing ECC from Argentina: In*900* (GenBank accession: KJ668594) in two ECC isolated in 2012 and 2013 in the Northern provinces of Salta (ST184) and Jujuy (ST749), respectively (15), and In883 (GenBank accession: KJ668593.1) isolated in 2011 in Chaco (ST182) (15). Here, In*900* was detected in Jujuy, Salta, Tucumán, Córdoba, and Buenos Aires City. Similarly, In*883* (ST182) was obtained in Chaco in 2016 from the same hospital as the previous isolate. These results suggest that, over time, the association of particular ECC-STs and In*900* or In*883* has managed to remain in the hospital environment over the years, particularly in the Northern provinces of the country (15). The molecular epidemiology of *bla*_IMP-8_ parallels that of VIM due to its frequent association with class 1 integrons. First detected in 2008 in an *E. cloacae* isolate (M9921) in Argentina, this gene was later found predominantly within the *Enterobacter* spp. group, reinforcing the strong association between this bacterial complex and IMP-8 (45, 46).

Accounting for the results obtained here, we can affirm that, even though VIM and IMP were the first carbapenemases associated with ECC, KPC-2 has become dominant at present, as detailed above, due to the potent regional dissemination of KPC (16, 40). Some authors argue that epidemiological scenarios with high burden of KPC-producing *K. pneumoniae* may influence the predominance of KPC over other enzymes in ECC as a consequence of (i) the emergence of epidemic carbapenemase-producing ECC clones stably associated with *bla*_KPC_-harboring plasmids (e.g., ST171); (ii) sporadic acquisition of diverse *bla*_KPC_-carrying plasmids by heterogeneous ECC clones; and (iii) the emergence of epidemic clones capable of harboring a variety of *bla*_KPC_-containing plasmids (e.g., ST78) (39). However, our findings challenge points (i) and (iii) and adhere more to the second hypothesis. Briefly, at the moment, we did not find a particular emerging epidemic clone in association with a specific carbapenemase type. The most abundant clones, such as ST88 and ST61, were associated with diverse carbapenemases, which is highly indicative of different plasmid locations. Moreover, ST88 and ST61 are not yet fit to be classified as high-risk or epidemic clones. ST171 has been shown to be associated with KPC in IncFIA in North America (39, 47). In our data set, none of the four ST171 isolates harbored KPC enzymes. Instead, these isolates carried class B and D carbapenemases and a double producer (NDM-1 + OXA-163).

Here, we analyzed the phenotypic behavior of the ECC isolates against those drugs not affected by the enzymatic activity of the carbapenemases. As a result, colistin, fosfomycin, and minocycline were the three most active drugs for KPC-, OXA-48-like-, and MBL-producing isolates, while CZA was the most active drug in KPC-producing ECC, colistin for OXA-producing ECC, and fosfomycin for MBL-producing ECC. It is well known that ECC is intrinsically resistant to penicillins and first- and second-generation cephalosporins due to low-level expression of chromosomal ampC genes encoding a cephalosporinase (46). Moreover, the constitutive hyperproduction (de-repression) of AmpC results in resistance to third-generation cephalosporins and aztreonam. Nowadays, with the progressive and rapid dissemination of resistance, ESBLs and AmpC-mediated resistance can typically be detected together, as observed in 37% of the isolates. Colistin resistance was identified in 13 isolates, of which only two (M22067 and M25166) harbored *mcr-9.1,* which could support the phenotypic result observed (see Tables S1 and S3 at https://github.com/sgomez-atb/Supplementary-Material-for-Genomic-Epidemiology-of-CP-ECC-in-Argentina-2016-22-; Fig. 2). Among these, in a single isolate (M27356), a mutation in *mgrB* (I4Yfs*7) that could explain the colistin resistance phenotype was found. In 11 isolates, missense mutations were found (see Table S2e at https://github.com/sgomez-atb/Supplementary-Material-for-Genomic-Epidemiology-of-CP-ECC-in-Argentina-2016-22-), but since there are no functional studies in the literature discussing these mutations, it is not possible to determine whether they are responsible for the observed colistin resistance phenotype. In addition, we found seven isolates that harbored *mcr-9.1* but showed susceptibility to colistin. This allelic variant of *mcr* was reported to be disseminated worldwide and has been extensively associated with *Enterobacter* spp. (21, 48–51). As mentioned in Results, resistance to colistin in *mcr-9*-positive bacteria is dependent on the simultaneous induction by colistin and the presence of the two-component system corresponding to a histidine kinase sensor (QseC) and its cognate partner (QseB) *mcr-9.1* (21). Here, we could not confirm the presence of *qseCB* because the *mcr* contigs were fragmented due to the sequencing method; therefore, long-read sequencing will be necessary to complete this issue.

To contextualize how the resistance determinants identified in this study influence therapeutic options in hospital settings, several factors must be considered: (i) carbapenemase class and co-occurring β-lactamases are key predictors of activity of newer β-lactam/β-lactamase inhibitor regimens (BL/BLI); (ii) metallo-β-lactamase producers are expected to compromise most BL/BLI combinations, often necessitating alternative strategies such as non-beta-lactam agents (colistin, fosfomycin, and tigecycline) or aztreonam-based approaches; and (iii) additional mechanisms, such as ESBL/AmpC overexpression and permeability/efflux alterations, may further erode efficacy even when a targeted inhibitor is available. These considerations reinforce the clinical value of timely, mechanism-directed diagnostics and support the use of this genomic surveillance data to inform local empirical therapy and infection prevention policies. Future work integrating harmonized clinical data—ideally through structured collaborations with infectious disease and stewardship networks—will be required to formally evaluate the impact of specific resistance mechanisms on treatment selection and patient outcomes

### Conclusions

The population structure of carbapenemase-producing *Enterobacter* spp. in Argentina is complex, involving the widespread dissemination of multiple species and subspecies across diverse clonal backgrounds. No consistent associations were observed between subspecies, sequence types, and specific carbapenemase profiles, reinforcing the prominent role of horizontal gene transfer in shaping resistance dynamics. Notably, *E. hormaechei* subsp. *steigerwaltii* accounted for half of the isolates, underscoring its substantial contribution to the national epidemiology of carbapenem resistance. These findings highlight a highly dynamic and evolving resistance landscape that requires continuous, high-resolution genomic surveillance to detect emerging clones and mobile genetic elements driving regional dissemination.

## MATERIALS AND METHODS

### Isolate selection and epidemiological data

The national surveillance network is composed of 93 representative clinical microbiology laboratories across the country connected through the WHONET network, in addition to the laboratories participating in the National Quality Assurance Program in bacteriology and antimicrobial resistance. In this context, all isolates suspected of having an emerging mechanism of resistance or that fall under the surveillance protocol are referred to the NRRLAR for further study. Between January 2016 and February 2022, 341 carbapenem-resistant ECC clinical isolates were referred by WHONET-network institutions to the NRRLAR for phenotypic and molecular characterization following specific local derivation guidelines for surveillance purposes. Species identification was completed using either traditional biochemical methods (*n* = 61) or MALDI-TOF MS (Bruker) (*n* = 70) in both cases, when available (see Table S1 at https://github.com/sgomez-atb/Supplementary-Material-for-Genomic-Epidemiology-of-CP-ECC-in-Argentina-2016-22-). For MALDI-TOF MS result interpretation, the Argentina RENAEM MALDI-TOF guidelines were used. These guidelines provide evidence-based criteria for genus- and species-level identification across 130 bacterial genera (22). Biochemical ID results were interpreted according to Bergey’s manual considering *E. cloacae* as follows (test [result]): triple sugar iron (acid/acid), citrate (positive), lysine decarboxylase (negative), ornithine decarboxylase (positive), hydrogen sulfide production (negative), indole (negative), and motility (positive) (see Table S1 at https://github.com/sgomez-atb/Supplementary-Material-for-Genomic-Epidemiology-of-CP-ECC-in-Argentina-2016-22-).

Suspected carbapenemase production was screened phenotypically according to the following local criteria (52): zone of inhibition to imipenem ≤22 mm for class A and B carbapenemases; ertapemen ≤24 mm; and piperacillin/tazobactam ≤15 mm for class D carbapenemases (see Table S1 at https://github.com/sgomez-atb/Supplementary-Material-for-Genomic-Epidemiology-of-CP-ECC-in-Argentina-2016-22-). Upon reception at the NRRLAR, 273 of 341 ECC isolates were confirmed as carbapenemase producers using an in-house multiplex PCR that includes *bla*_KPC_, *bla*_IMP_, *bla*_NDM_, *bla*_VIM_, and *bla*_OXA-48-like_. The primers and protocol are in the public domain (https://antimicrobianos.com.ar/ATB/wp-content/uploads/2021/01/Detección-CBP-Multiplex.pdf). To perform this study, a representative sample of 131/273 CP-ECC was selected, considering one isolate per patient, at least one isolate per province, one isolate per institution, and one isolate of each carbapenemase type and/or combination of carbapenemase per institution. Isolates that were obtained from rectal swabs or that were part of a presumed or confirmed outbreak were excluded.

The 131 isolates were obtained from 68 institutions located in 17 of 24 jurisdictions throughout the country (see Fig. S1 and Table S2a at https://github.com/sgomez-atb/Supplementary-Material-for-Genomic-Epidemiology-of-CP-ECC-in-Argentina-2016-22-). The majority of the isolates were obtained from two jurisdictions, Buenos Aires City (CABA) (*n* = 40; 30.5%) and Buenos Aires Province (*n* = 24; 18.3%). The median age of the patients was 49 years, with a range of 10 days to 86 years of age. The sex distribution was similar in males (*n* = 71; 54.2%) and females (*n* = 60; 45.8%). The most frequent sample type was urine (*n* = 43; 32.9%), followed by blood (*n* = 41; 31.3%), abdominal fluid (*n* = 11; 8.4%), skin and soft tissue (*n* = 8; 6%), tracheal aspirate (*n* = 5; 3.8%), and bone (*n* = 4; 3%), among other sample types (see Tables S1 and S2a at https://github.com/sgomez-atb/Supplementary-Material-for-Genomic-Epidemiology-of-CP-ECC-in-Argentina-2016-22-).

### Antimicrobial susceptibility testing

Antimicrobial susceptibility testing was performed by the disk diffusion method following Clinical and Laboratory Standards Institute (CLSI) recommendations. Susceptibility to aztreonam, amikacin, gentamicin, ciprofloxacin, trimethoprim/sulfamethoxazole, and minocycline was interpreted according to the Clinical and Laboratory Standards Institute (Table 1; see Table S1 at https://github.com/sgomez-atb/Supplementary-Material-for-Genomic-Epidemiology-of-CP-ECC-in-Argentina-2016-22-) (53). Fosfomycin and CZA susceptibility was interpreted with the European Committee on Antimicrobial Susceptibility Testing (EUCAST) breakpoint (54). The colistin testing methods used were colistin agar spot and colistin drop test, following the procedures previously reported and EUCAST interpretation criteria (55). The susceptibility results of the 131 isolates were analyzed using WHONET 5.6 software (https://whonet.org/) to obtain cumulative susceptibility reports according to CLSI recommendations (56).

### Whole-genome sequencing and bioinformatics analysis

Whole bacterial DNA was extracted with QIAcube, using the QIAamp DNA Blood Mini Kit (Qiagen, Hilden, Germany), and sequencing was performed using the Nextera XT DNA library preparation kit. MiSeq system (Illumina, CA, USA) was used to generate 150-bp paired-end reads. Short reads were trimmed and quality filtered (Phred score ≥30) with Trimmomatic v0.39 (57) and assembled with Unicycler v0.5.0 (58). MLST was identified with Ariba v2.14.6 (PubMLST database), and resistance genes and point mutations were identified with ResFinder v4.6.0 and AMRFinderPlus (59) v3.11.26 with database v2023-11-15.1. Additionally, colistin resistance was manually evaluated in the two-component systems *phoP/phoQ*, *pmrAB*, and its negative regulator, *mgrB*, by comparing the DNA sequence in susceptible strains with resistant isolates from our panel. The close genetic environment of carbapenemase genes was graphed using https://proksee.ca/.

### Taxonomical evaluation and species and subspecies identification

Species identification was performed with the Speciator tool (60) included in Pathogenwatch. Additionally, ANIclustermap software was used to make a UPGMA hierarchical clustering using an all vs all matrix of the average nucleotide identity values calculated with fastANI (61). An ANI threshold of 94% was considered to categorize species and 98% for subspecies using reference genomes (23). The clustermap was obtained with ANIclustermap 1.2.0. Table S6 includes all the reference genomes used.

### Phylogenetic analysis

To build the pan-genome phylogenetic tree, 12 complete reference *Enterobacter* genomes belonging to each species and subspecies were selected and downloaded from NCBI (23). Once downloaded, the reference genomes were re-annotated together with the clinical strains using Bakta v1.9.1 with database v5.0 (62). Roary v3.12.0 (63) was used to generate a core gene alignment (concatenated genes present in >99% of the genomes with >95% of nucleotide identity) with all genomes annotated with Bakta software. The core alignment was used to generate a SNP alignment with SNP sites v2.3.3 (64) . A maximum likelihood tree was constructed using RAxML (65)v8.2.11 under the GTR model with the gamma distribution to model site heterogeneity (GTRGAMMA) using 1,000 bootstrap replicates with the SNP core alignment. The phylogenetic tree was plotted with iTOL v6.9.1 (66).

### Statistical analysis

Statistical differences were determined using 2×2 contingency tables in the GraphPad calculator. Statistically significant differences were considered with *P* < 0.05.

## Data Availability

All data presented in this document are freely available in this text or in the supplemental material deposited in GitHub: https://github.com/sgomez-atb/Supplementary-Material-for-Genomic-Epidemiology-of-CP-ECC-in-Argentina-2016-22-.git. The sequence data have been deposited and are available in GenBank under BioProject PRJNA1159154.
